# Subacute duodenal obstruction caused by Common Celiaco-Mesenteric Trunk anomaly-A case report

**DOI:** 10.1016/j.ijscr.2021.106043

**Published:** 2021-05-26

**Authors:** Priyanka Saha, Keerthika Reddy Rachapalli, Rajeshwari Bhat B, Waqar Ahmed Ansari, Asif Ansari, Hridayanath Desai

**Affiliations:** Department of General Surgery, Grant Government Medical College and Sir J&J Group of Hospitals, Mumbai, India

**Keywords:** CA, celiac artery, CMT, celiac mesenteric trunk, SMA, superior mesenteric artery, LGA, left gastric artery, CHA, common hepatic artery, SA, splenic artery, CECT, contrast-enhanced computed tomography, BMI, body mass index, Common celiacomesenteric trunk, Duodenal obstruction, Wilkie's syndrome, Duodenojejunostomy, Mesenteric vascular anomalies, Case report

## Abstract

**Introduction and importance:**

The origin of the mesenteric vasculature is highly variable. One such variation is the common celiaco-mesenteric trunk (CMT). To our knowledge, this is the first reported case of subacute duodenal obstruction caused by common CMT. The awareness of this anomaly helps keep a high index of suspicion for varied presentations, prompts appropriate investigations, timely intervention, and avoids iatrogenic injury.

**Patient profile:**

A 15-year-old boy presented with a history of repeated attacks of colicky abdominal pain with bilious vomiting. Computed tomography of the abdomen with intravenous contrast revealed subacute duodenal obstruction caused by an acute angulation of common CMT with the abdominal aorta. To relieve the obstruction, the patient underwent a side-to-side duodenojejunostomy.

**Discussion:**

A common CMT, where the coeliac artery (CA) and superior mesenteric artery (SMA) have a common origin from the aorta, accounts for less than 1% of all splanchnic artery anomalies. Most CMTs are incidental findings, but aneurysm or dissection of the common trunk commonly accompany this anatomical aberrancy. Intestinal obstruction due to CMT anomaly is a rare occurrence.

**Conclusion:**

There should be a high index of suspicion concerning vascular anomalies in patients, especially children presenting with repeated episodes of subacute intestinal obstruction. This knowledge of vascular aberrations prevents disastrous iatrogenic complications.

## Highlights

• Rarity of the occurrence of common celiacomesenteric trunk
• As per our knowledge this is the first reported case of CMT presenting with duodenal obstruction.
• Its similarity to Wilkie's syndrome in terms of patient and management
• Different presentations of CMT
• Knowledge of this variations important in other surgeries and endovascular procedures to avoid dreadful complications.

## 1 Introduction

The celiac artery (CA) and the superior mesenteric artery (SMA) arise ventrally from the abdominal aorta. Several anatomical variations are described in literature. One such variation is the common celiaco-mesenteric trunk (CMT). These variations are due to aberrations in the embryological development of the four ventral splanchnic arteries arising from the primitive abdominal aorta, i.e., the left gastric artery (LGA), common hepatic artery (CHA), splenic artery (SA), and SMA [1]. This anomaly is commonly asymptomatic. But mesenteric ischemia due to thrombosis or injury or aneurysm of CMT, nutcracker syndrome due to compression of renal artery by CMT have been reported.

This article reports a case of subacute intestinal obstruction caused by a common CMT anomaly, investigated and managed at our tertiary care teaching hospital. After a thorough literature review, there have been no reports on subacute duodenal obstruction caused by a common CMT anomaly to date. This work has been reported in line with SCARE criteria [2].

## 2 Case report

A 15-year-old male patient presented to the emergency department with features of subacute intestinal obstruction. The patient complained of intermittent episodes of abdominal pain, distension and bilious vomiting since childhood which were relieved spontaneously or with over-the-counter medications. With a BMI of 16 kg/m^2^, the patient also reported an inability to gain adequate weight. The abdominal examination was unremarkable.

**Fig. 1:**
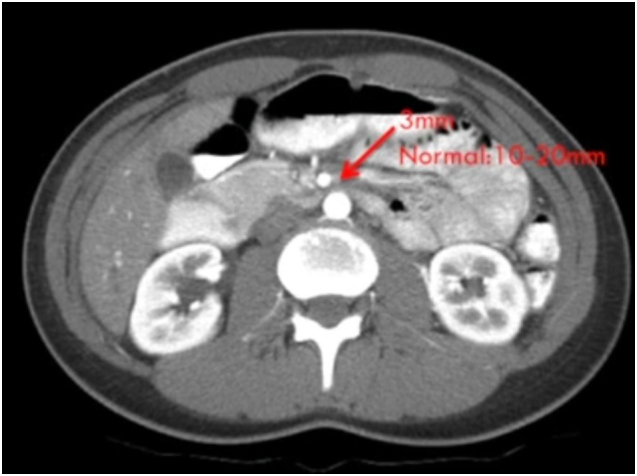
CECT abdomen showing vascular loop compressing the third part if duodenum with proximal dilatation.

**Fig. 2:**
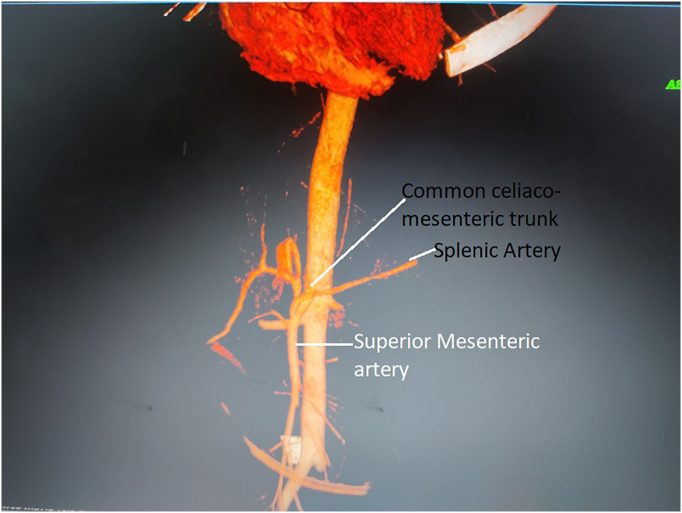
3-D reconstructed image of the CT angiography showing common origin of the CA and SMA, origin of the SA from the SMA, narrow angle between the CMT and abdominal aorta.

Erect X-ray and ultrasonography of the abdomen were unremarkable. Contrast-enhanced computed tomography (CECT) with angiography showed extrinsic obstruction of the third part of the duodenum with resultant dilatation of the stomach and duodenum (maximum diameter 4.3 cm). CA and SMA had a common origin from the abdominal aorta at the lower border of L1, forming a common CMT. CMT formed an angle of 19 degrees with the aorta compressing the third part of the duodenum. 3D reconstruction of the images confirmed the findings mentioned above while providing detailed anatomy of the CMT (Fig. 1, Fig. 2).

**Fig. 3:**
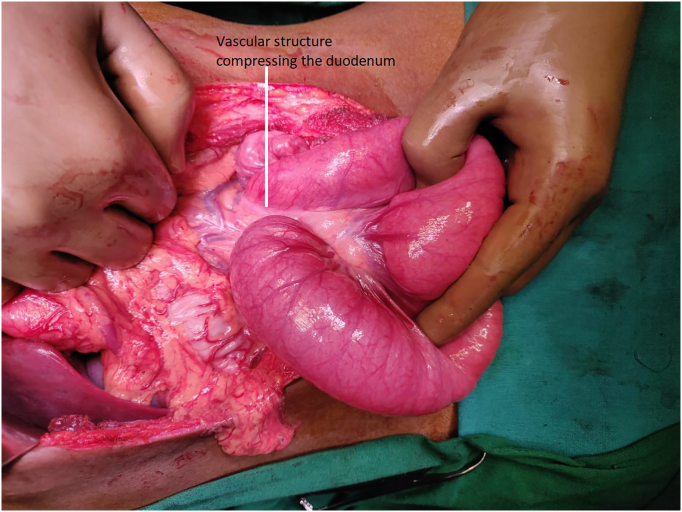
Showing compression of the third part of the duodenum by CMT (marked by a white arrow).

The patient was optimised nutritionally and taken up for exploratory laparotomy. Intra-operatively, the stomach, first and second parts of the duodenum were dilated and hypertrophied. There was an abrupt cut-off in the dilated duodenum where the CMT crossed it. These findings confirmed the diagnosis (Fig. 3).

**Fig. 4:**
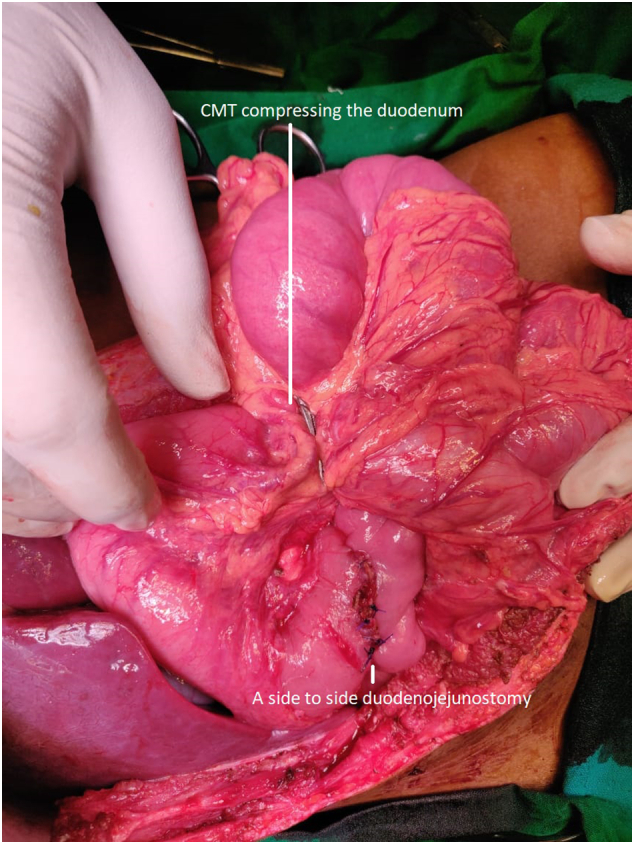
Showing a side-to-side duodenojejunostomy.

A side-to-side duodenojejunostomy was performed to bypass the obstruction. The post-operative period was uneventful, and the patient obtained symptomatic relief (Fig. 4).

Follow-up at 3, 6 and 12 months showed an improved BMI of 17.8 kg/m^2^. He had no recurring symptoms and was pain-free thereafter.

## 3 Discussion

**Fig. 5:**
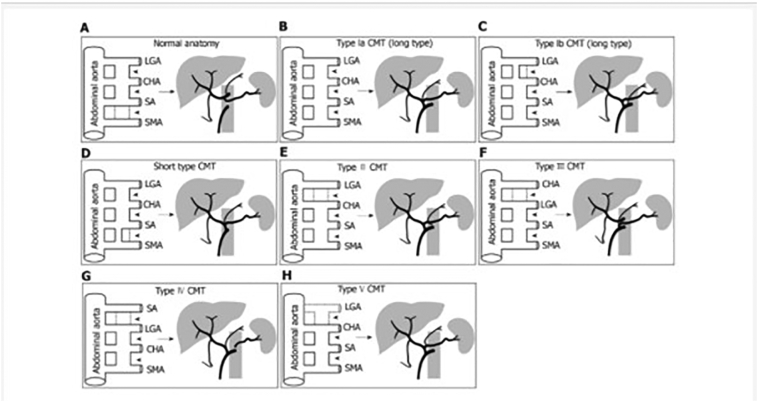
Schematic diagram of embryological development for normal anatomy and celiacomesenteric trunk variants of the celiac trunk and the superior mesenteric artery A: when the longitudinal anastomosis is interrupted between roots 3 and 4, the normal configuration of the CA and SMA is generated; aberrations in this lead to many variations, as mentioned below: B: persistence of the longitudinal anastomosis among all four roots generate a type Ia celiaco-mesenteric trunk (CMT); C: an incomplete interruption between roots 1 and 2 results in type Ib CMT. Both type Ia and Ib are long CMT. D: An incomplete interruption between roots 3 and 4 results in a short CMT; E: interruption between roots 1 and 2 generates a type II CMT. F: If root 1 is the CHA, the interruption occurs between roots 1 and 2, results in a type III CMT. G: If root 1 is the SA, the interruption between roots 1 and 2 results in type IV CMT; H: type V is due to complete regression or absence of the root 1, a replaced LGA arising from other arteries [3].

The primitive dorsal abdominal aorta gives origin to four ventral mesenteric roots- LGA, CHA, SA, and SMA, connected by a longitudinal anastomosis (Fig. 5) [3].

The interruption in the longitudinal anastomosis between SA and SMA generates the normal configuration of CA and CMA. Any deviations in these lead to the formation of different types of common CMT [3].

Common CMT, a rare anatomical variant, has a common origin of the CA and SMA from the aorta, with an estimated incidence of 0.25% [4,5]. In this patient, the common trunk gave rise to CHA, LGA, and SMA, and the SA originated from the proximal SMA. The common CMT formed an angle of 19 degrees with the aorta, compressing the third part of the duodenum.

This anomaly is associated with clinical conditions like compression of CMT by abdominal aortic aneurysm [6], gastrointestinal infarction due to thrombosis of the CMT [7], nutcracker syndrome [8], gastrointestinal ischemia due to aortic dissection extending to the CMT [9]. To date, this is the first reported case of a common CMT presenting as a subacute duodenal obstruction, mimicking Wilkie's syndrome.

Von Rokitansky first described the compression of the anterior duodenal wall by the SMA as a cause of duodenal obstruction in 1861 on a post-mortem case. It was studied in detail by D. Wilke in 1912 [10]. This vascular compression of the duodenum received many names over the years, such as SMA syndrome (arterio-mesenteric duodenal compression syndrome, the cast syndrome, and chronic duodenal ileus). The reported prevalence in the general population varies between 0.013% and 0.78% [11].

The length and attachment of Treitz's ligament, the level at which the duodenum crosses the vertebral column, influences the angle of SMA origin [11]. The normal SMA-aorta angle of origin ranges between 20° to 70°, whereas in Wilkie's syndrome, it steeply ranges from 6° to 15° [12]. There has been no mention of the normal angle made by the common CMT with the aorta in literature. In this patient, the narrow-angle (19°) of origin of the common CMT obstructed the third part of the duodenum.

The primary symptoms of such vascular compressions are epigastric pain and vomiting, progressively increasing in severity, causing fluid and electrolyte disturbances with weight loss. Radiological imaging confirms the diagnosis [13]. CECT abdomen will show the distended first, second, and third parts of the duodenum with the demarcation of the obstructed lumen by the vasculature [14,15], and excludes other causes of duodenal obstruction (tumors, annular pancreas). Colour Doppler ultrasound and CT angiography help measure the angle of origin of the mesenteric vasculature. Therefore, the investigation of choice remains CECT of the abdomen with angiography.

The operative options include duodenojejunostomy, transection of the ligament of Treitz and relocation of the duodenojejunal junction (Strong's Procedure), gastrojejunostomy and anterior duodenal replacement [16]. Starley first introduced Duodenojejunostomy in 1910, and over the years, it has become the most frequent treatment with a success rate of 90%.

Owing to the anatomical similarities, a duodenojejunostomy can be performed in a common CMT causing duodenal obstruction as done in our case.

## 4 Conclusion

An understanding of the clinical relevance of CMT anomaly remains anecdotal, given its infrequency. Nevertheless, most incidentally diagnosed CMTs without intrinsic disease will remain asymptomatic. In patients presenting with repeated episodes of subacute intestinal obstruction, especially in the pediatric age group, there should be a high index of suspicion concerning vascular anomalies such as these. CECT abdomen with angiography remains the investigative modality of choice. The awareness of this anatomical variation helps plan therapeutic options and reduce the chance of surgical injuries.

## Ethical approval

Our study is exempt from ethical approval.

## Funding sources

This research did not receive any specific grant from funding agencies in the public, commercial, or not-for-profit sectors.

## CRediT authorship contribution statement

Dr. Priyanka Saha was the assistant operating surgeon, helped perform literature review and condense the manuscript to meet author guidelines.

Dr. Keerthika Reddy helped draft the operative section of the manuscript and performed literature review.

Dr. Rajeshwari Bhat B helped draft the manuscript discussion, edited images and is the corresponding author.

Dr. Waqar Ansari was the lead surgeon on the case, helped draft the manuscript and reviewed it prior to submission.

Dr. Asif Ansari was assistant operating surgeon and performed a literature review.

Dr. Hridayanath Desai was the assistant operating surgeon and reviewed it prior to submission.

## Guarantor

Dr Priyanka Saha.

## Research registry

Research registry unique identification number: researchregistry6675.

## Consent

A written informed consent was obtained from the patient and relatives for the publication if this case report and images. A copy of this available for review by the Editor-in-Chief of this journal.

## Declaration of competing interest

No conflicts of interest between the authors.
